# Building the Systematic Review Core in an academic health sciences library

**DOI:** 10.5195/jmla.2019.711

**Published:** 2019-10-01

**Authors:** Mellanye J. Lackey, Heidi Greenberg, Melissa L. Rethlefsen

**Affiliations:** Health Sciences Library, University of Nevada, Las Vegas, Las Vegas, NV, mellanye.lackey@unlv.edu; Spencer S. Eccles Health Sciences Library, University of Utah, Salt Lake City, UT, heidi.greenberg@utah.edu; Health Science Center Libraries - George A. Smathers Libraries, University of Florida, Gainesville, FL, mlrethlefsen@gmail.com

## Abstract

**Background:**

The authors present efforts to build capacity at our institution for conducting systematic reviews and other forms of evidence synthesis through partnerships and a recharge model. This report describes how we successfully created and launched a for-fee systematic review core at our library.

**Case Presentation:**

Throughout 2014 and 2015, library leadership proposed different models for getting institutional and financial support for librarians and staff to better support university researchers conducting systematic reviews. Though well received, initial requests for financial support were not funded. The executive director of the Health Sciences Library released two years’ worth of salary and benefits to fund an evidence synthesis and retrieval librarian position. With this new position, the team formed a charge-back core facility in partnership with our university’s Clinical Translation and Science Award hub. A series of procedural decisions and operational changes helped the group achieve success. Within eighteen months after launching the Systematic Review Core, we reached maximum capacity with more than twenty ongoing reviews.

**Discussion:**

Assigning a dollar value to our expertise put us on par with other subject matter experts on campus and actually drove demand. We could act as paid consultants in research projects and shifted the perception of librarians from service providers to research partners. Affiliating with our partners was key to our success and boosted our ability to strengthen our campus’ research infrastructure.

## BACKGROUND

Publishing high-quality accurate systematic reviews (SRs) with reproducible, well-described methods is an increasing priority for researchers [1, 2]. Librarians and information specialists are key to achieving reproducible, high-quality SRs and knowledge syntheses [3–7]. Increasingly, librarians perform essential components of SRs, including planning, executing, and reporting the search methodology and results [8]. All of these contributions can take significant time [9, 10] as well as require extensive knowledge of the latest methods and techniques in expert searching and knowledge synthesis methodology and typology [7, 8].

Many service models for SR services have emerged at health sciences libraries and other academic libraries as demand for librarian involvement has increased over the past five to ten years. Service models vary significantly by library. Some libraries have dedicated SR services, comprising a librarian or librarians whose main role is to contribute to SRs [11]. More commonly, libraries have teams of librarians with small portions of their duties dedicated to performing SRs [12–15]. Other libraries with liaison models encompass SRs into standard liaison librarian duties and do not have special services [16, 17]. Significant work has been done to showcase successful models of SR programs at various health sciences libraries through professional organizations, including the Medical Library Association, as communities began to expect that librarians and information specialists were required for successfully conducting SRs.

Though many libraries have established models for successfully developing an SR service or SR support, these services and support tend to be library-centric and are funded and promoted by the libraries themselves. Libraries that charge for in-depth SR services or support tend to follow a tiered service structure, in which the price increases with the level of service provided [18, 19]. Libraries’ fee-based service models generally exist outside the overall institutional research services infrastructure or “core facilities” model, though they share many commonalities.

Core facilities often utilize fee-based models for cost recovery, which may also be called “recharge” units or service centers. The National Institutes of Health (NIH) describes core facilities as:

centralized shared research resources that provide access to instruments, technologies, services, as well as expert consultation and other services to scientific and clinical investigators. The typical core facility is a discrete unit within an institution and may have dedicated personnel, equipment, and space for operations. [20]

Fee structures for these core facilities and recharge centers are governed by the Office of Management and Budget Guidance and must only be used for cost recovery, not profit [21]. The slate of core facilities at institutions greatly impacts their researchers’ ability to do cutting-edge research by providing access to expanded, centralized research resources like flow cytometry, X-ray crystallography, or proteomics [22]. No institution to the authors’ knowledge has yet utilized a formal “core facility” or NIH recharge model to provide specialized library services, though such services clearly could fall within the scope of the NIH definition.

At the University of Utah Spencer S. Eccles Health Sciences Library (EHSL), we faced a challenge: the desire to provide SR services with limited capacity to move forward both in terms of staff and faculty expertise and time. We did have numerous advantages, however, including a close relationship with the Center for Clinical & Translational Science (CCTS), established through many joint strategic efforts that were designed to increase the capacity for research on campus [23]. In addition, the university had established multiple core facilities and had already consented to include the EHSL’s Research Information Services on the main University of Utah Health Sciences Center (HSC) Cores website. In addition, the CCTS had already established fee-based service groups, including the Study Design & Biostatistics Center, which provides consultation and in-depth statistical work. Building upon these factors, we created capacity for conducting and supporting SRs and other forms of evidence synthesis at the EHSL, using a core facilities model in partnership with our CCTS.

## CASE PRESENTATION

In early 2014, one staff member with many years of experience as an expert searcher was the library’s only person working with SR teams. She created complex, in-depth searches for SRs, while balancing other demands and responsibilities in her full-time position but was unable to offer broader consultative services on SR methodology. Beginning in 2014, a new library faculty member, who also had experience and interest in conducting SRs, highlighted that support for SRs could be a major strategic consideration for the EHSL.

Interested librarians and staff started offering additional SR services in a non-centralized manner. Because each librarian worked independently on projects, communications with the project teams were not standardized. Methodological write-ups varied depending on the librarian’s expertise, capacity, and willingness to follow-up amidst competing work priorities. Requests for assistance with SRs came through several different channels. Search strategies were informally peer reviewed, and time spent on projects was estimated, not tracked. Librarian and library staff involvement in SRs was seen as a service, rather than a partnership, and overall, there were low levels of awareness on campus of the expertise available for this type of research. Nevertheless, the number of requests was increasing, as was our interest in providing a higher quality product.

Throughout 2014 and 2015, library leadership proposed different models for getting institutional and financial support for the EHSL to better support university researchers who were conducting SRs. We initially proposed that the CCTS would fund us to hire one full-time equivalent (FTE) librarian (a faculty position) who would focus on creating search strategies, project management, and information management for SRs. The proposal also requested budget for the cost of Embase, which we considered essential for high-quality evidence synthesis. The librarian would also create education and training materials for those who wanted to learn more about SRs, thus boosting the research acumen of the health sciences campus and the larger university community. The CCTS was very excited about the idea of partnering with their library colleagues to create an SR service, but their budget did not contain funds to cover a faculty position, especially without knowing if the uptake of SR services would justify the cost.

To mitigate these challenges, the EHSL executive director decided to strategically release two years’ worth of salary and benefits to fund an evidence synthesis and retrieval (ERS) position. First, we wanted to demonstrate to the university’s researchers and administrators how librarian expertise would benefit SRs. Second, we wanted to build a salary reserve to sustain this position. The creation of this position, fully paid from the library’s budget for two years, enabled us to renegotiate with the CCTS—and the CCTS enthusiastically responded.

Though the CCTS was unable to offer funding, they did pledge partnership, support, and assistance to us to create a new core within the CCTS. Specifically, we partnered with the Population Health Research Foundation for Discovery (PHR), a CCTS team consisting of four cores or “sections”: Study Design and Biostatistics Core; Health Economics; Qualitative, Survey and Measurement Core; and Cancer Biostatistics Shared Resource. The Systematic Review Core (“SR Core”) became the fifth core in this group, and all of the cores used the same request, billing, and chargeback systems and structurally reported through the same PHR leaders. All of the core section directors, the heads of the particular cores, met together biweekly to ensure similarity of processes and collaboration.

The founding section director was the ESHL’s deputy director, who authored the prior proposals to the CCTS and was an experienced systematic reviewer with a strong interest in methodology and innovation in review methods. She was joined by two existing library team members with extensive experience in SRs and SR methodologies. This included the expert searcher, who had sustained SR efforts to this point, and the associate director for education and research, who had experience working with many project research teams on their SRs. She also had an interest in growing the skills and knowledge of other EHSL librarians so that they could actively partner on SR teams. The fourth team member would be the new ERS librarian, who began in July 2016, seven months after the official launch of the SR core.

Initially, we set a rate of $103 per hour for librarian expertise for SRs, meta-analyses, and other forms of evidence synthesis. This rate was comparable to rates the other cores charged for services performed by faculty with master’s degrees, such as statisticians or survey designers. Any monies received from our clients would return to the library to fund the ERS librarian’s salary. To further supplement the position, the ERS librarian, when hired, was expected to seek out partnerships with researchers or project groups who would include the librarian’s time in grants and contracts.

To prepare for the launch of the SR Core, we made several process and operational decisions. We decided that all groups who requested help would receive a free two-hour consult with a member of our team, similar to policies established by the other CCTS cores. During the consult, we would help them refine their research questions, educate them about the SR process, encourage them to draft and publish a protocol, and assess their readiness to begin an SR or a scoping review.

We decided that we would not create search strategies for groups in those initial two hours. We also decided that if a project used the expertise of the SR Core, then the team member who worked with that project would write the methods section for the manuscript. As a contributing author, they would then get authorship on any publications or presentations about the review, including the protocol, if applicable, and the final paper.

After the initial consult, if we established that project teams needed in-depth search support but neither SRs nor scoping reviews, we decided that we would refer them to the Reference and Instruction Team at the library. If project teams were undertaking an evidence synthesis project but were unable to pay to use the services of the SR Core, then we would direct them toward resources such as the *Cochrane Handbook*, online tutorials and guides, books about conducting SRs, SR LibGuides, and other documentation to help them learn about the process and requirements of conducting evidence syntheses.

Once the SR Core was fully operational, we recognized that with four team members working on several reviews simultaneously, we needed consistency in our information management. We started by using Google Drive to share and store files and track projects but, after several months, transitioned to Box, the campus-supported online cloud storage and collaboration tool. We developed a common file structure and naming conventions in Box to organize project documents, EndNote libraries, search strategies, and other notes. We developed standard verbiage and templates for communications with clients. We also developed a template for writing protocols and manuscript methodologies to ensure consistency and quality across projects. The increased organization helped us streamline the process from conducting intake interviews to submitting final manuscripts in a way that was thorough and efficient.

We also aimed for efficiency in our workflows as we created search strategies, translated them to different databases, and de-duplicated citations for review by project teams to maximize our time in order to be cost effective. We sought meaningful training opportunities that would increase efficiency, such as incorporating Bramer et al.’s method of using macros to create and translate search strategies [24]. We also used the PRESS guidelines for peer review to formalize our efforts [25]. Because acting as a CCTS core also required contributing to development of innovative methods, we conducted research as we strove to improve our own methods.

Over the first year of the SR Core’s existence, major changes happened with the CCTS’s governance and structure, including a transition to more stringent recharge center policies. This meant moving several of the cores from a departmental subsidization model to an hourly charge model. For the SR Core, this meant a reanalysis of our hourly charge rates. Critical to this analysis was the amount of time each member of the core was expected to contribute to the SR Core. We based our rates on the ERS librarian working full time (1 FTE) on reviews, the expert searcher working 30 hours per week (0.75 FTE), and the associate director and deputy director taking on 1–2 reviews a year, as time allowed (0.1 FTE each). The associate and deputy directors additionally performed administrative management duties for the SR Core. In addition, to establish a true picture of costs as allowed by the Office of Management and Budget Guidance, we included a portion of the costs for our Embase subscription, as well as student staff hours for completing interlibrary loan requests on behalf of our clients (10 hours per week). The new established rate lowered our costs to $93 per hour beginning July 1, 2017.

Within eighteen months of the launch of the SR Core, we determined that we had reached maximum capacity for taking on new projects, with more than twenty ongoing reviews. Collectively, we had earned over half of a year’s salary to sustain funding for the ERS librarian. More importantly, we became fully integrated into the campus research infrastructure.

## DISCUSSION

When we began the SR Core, we had trepidations about how our community would react to being charged for a previously free service. Our early conversations exploring issues of library mission—tradition, sustainability, and relationships with users—echoed decades of debate about charging fees for library services [26]. We discussed in depth what constituted an integrated library service as opposed to a service that required time and expertise beyond the scope and capacity of the service model that we used at the time. In particular, we were uncertain whether the research groups that had used the expertise of the library without paying previously would be willing to pay for the service. Contrary to our fears, assigning a dollar value to our expertise put us on par with other subject matter experts on campus and actually drove demand. We could easily act as paid consultants in research projects, for example, whereas this potential was previously overlooked. Research groups had always been able to include a librarian’s time on their grants, but CCTS and institutional support elevated the status of our expertise so that research groups increasingly requested partnerships with the SR Core.

We also recognized this change would impact unfunded SR projects. We wished that we could offer every group adequate and appropriate support for their research, regardless of their ability to pay, but the recharge center model we embarked on did not allow us to partner with all interested teams. More to the point, not charging for our expertise would undercut the ability to fund the ERS librarian salary in the long term. Forcing ourselves to use this model became a great advantage for us and for our clients. We charged for librarian expertise that offered in-depth, priority focus for SRs. High-level evidence synthesis no longer competed for time with all the other duties that the team members were performing, which meant the reviews could be done more quickly and with higher-quality results.

The SR Core’s affiliation with the PHR was critical to its success on multiple levels and, ultimately, what distinguished us from the SR service models at previously mentioned HSLs [11–17]. For one, the partnership aligned us with a high-profile group in the CCTS. Our group now existed under the larger umbrella of an influential organization rather than existing only in the library. As a PHR core, we were invited to attend PHR all-staff meetings. In those meetings, we participated in discussions with other cores’ members, taking every opportunity to promote our expertise and capacity to partner on SRs. Meeting attendees were initially surprised at the breadth of knowledge that librarians had about conducting SRs and our possible roles in SR projects, as described in the scoping review by Spencer and Eldredge [8].

The increased visibility of the SR Core at the meetings led to an increase in awareness about our expertise, which in turn led to an uptick in requests to join SR projects. This also enabled us to identify key methodologists, especially statisticians, whom we could bring into meta-analyses and other methodologically complex projects. While promotion and the sense of community in the PHR were key advantages, logistical support was essential. The PHR already subscribed to a time-tracking system to record the number of hours spent on each project and produce reports of billable hours to invoice departments on campus. The SR Core members submitted time sheets weekly through the system and then received monthly and quarterly reports detailing the number of hours each person worked on each project that clearly enumerated our involvement on campus research. Helpfully, the PHR administrative staff handled invoicing and billing on our behalf.

We still required internal assistance from our own finance and administrative staff, especially to help us process grant funds and strengthen procedures and documentation. The internal assistance was essential to tracking our progress across multiple, simultaneous projects and substantially improved communications in the core. This cost was borne by the EHSL and not recovered. We recorded any time spent on reviews, including background research, protocol development, search strategy development and testing, citation de-duplication, and set up of project tools like Covidence.

By tracking how much time was spent by whom and on which review, we had accurate data about what we as librarians spent our time on and at what cost. Bullers et al. note that librarians in academic settings often underreport time spent or underestimate the total time spent, which makes administrative planning a challenge [9]. We have used the data to inform planning and budgeting decisions for the SR Core. It informed our decision about how many simultaneous reviews we could manage as well as when we would be available for starting new projects. Our tracking data helped us to predict how much time we would spend on reviews—vital information for our partners to include in their budget estimates and plans.

The SR Core strengthened the role of the EHSL in our campus’s research infrastructure. Previous studies have found that librarian involvement in SRs correlated with higher quality reporting of search strategies [3–6]. Following our own findings, we had a strong commitment to consistent and precise reporting of the search strategies, databases with descriptions, and the search processes in every review so that results of the reviews would be more reproducible. The SR Core also improved our ability to meet campus research needs. The success of the SR Core was perhaps most clearly demonstrated in the EHSL’s budget. Our advocacy, participation in meetings, and data, along with a demonstrated interest in collaboration, shifted expectations in the PHR and among campus administrators about our value as SR partners.

In 2017/18, we received centrally funded start-up money to hire a new faculty member who would conduct research on methods in evidence synthesis. This start-up funding was the first ever given to a library on the University of Utah campus. Our eventual goal was to increase research and teaching capacity and become a center of excellence for SR methodology. However, personnel changes forced us to shift our plans for the future. All three faculty team members took on new opportunities and new positions elsewhere.

Coupled with recruitment challenges, in May 2018, we collectively decided to close the SR Core until it could be revived. As of mid-2019, the SR Core has begun re-establishing its role in the CCTS PHR with the expert searcher staff member as the new section head. A new faculty member has been recruited to the team, and we anticipate its growth back into a successful enterprise with continued support and encouragement from our CCTS colleagues. One of us took this model to the University of Florida, where she is building on our previous successes with a new CTSA hub–health sciences library partnership. Another one of us hopes to bring the model to the University of Nevada, Las Vegas, as the university expands its research capacity. We encourage other SR teams at libraries to consider creating their own versions of the SR Core to fund librarian participation in SR projects.

## GRANT SUPPORT

The project reported in this publication was supported in part by the National Center for Advancing Translational Sciences of the National Institutes of Health under award number **UL1TR002538**. The content is solely the responsibility of the authors and does not necessarily represent the official views of the National Institutes of Health.

## 

**Mellanye J. Lackey, AHIP**, mellanye.lackey@unlv.edu, https://orcid.org/0000-0003-0574-7894, Health Sciences Library, University of Nevada, Las Vegas, Las Vegas, NV

**Heidi Greenberg**, heidi.greenberg@utah.edu, Spencer S. Eccles Health Sciences Library, University of Utah, Salt Lake City, UT

**Melissa L. Rethlefsen, AHIP**, mlrethlefsen@gmail.com, https://orcid.org/0000-0001-5322-9368, Health Science Center Libraries - George A. Smathers Libraries, University of Florida, Gainesville, FL
